# Virtual reality assisted microscopy data visualization and colocalization analysis

**DOI:** 10.1186/s12859-016-1446-2

**Published:** 2017-02-15

**Authors:** Rensu P. Theart, Ben Loos, Thomas R. Niesler

**Affiliations:** Stellenbosch University, Stellenbosch, 7600 South Africa

**Keywords:** Virtual reality, Hand tracking, Colocalization analysis, Region of interest selection, Confocal microscopy visualization, 3D Microscopic reconstruction, Volume rendering

## Abstract

**Background:**

Confocal microscopes deliver detailed three-dimensional data and are instrumental in biological analysis and research. Usually, this three-dimensional data is rendered as a projection onto a two-dimensional display. We describe a system for rendering such data using a modern virtual reality (VR) headset. Sample manipulation is possible by fully-immersive hand-tracking and also by means of a conventional gamepad. We apply this system to the specific task of colocalization analysis, an important analysis tool in biological microscopy. We evaluate our system by means of a set of user trials.

**Results:**

The user trials show that, despite inaccuracies which still plague the hand tracking, this is the most productive and intuitive interface. The inaccuracies nevertheless lead to a perception among users that productivity is low, resulting in a subjective preference for the gamepad. Fully-immersive manipulation was shown to be particularly effective when defining a region of interest (ROI) for colocalization analysis.

**Conclusions:**

Virtual reality offers an attractive and powerful means of visualization for microscopy data. Fully immersive interfaces using hand tracking show the highest levels of intuitiveness and consequent productivity. However, current inaccuracies in hand tracking performance still lead to a disproportionately critical user perception.

**Electronic supplementary material:**

The online version of this article (doi:10.1186/s12859-016-1446-2) contains supplementary material, which is available to authorized users.

## Background

In the past, biological visualization has been limited to rendering on two-dimensional displays. The use of three-dimensional displays for quantitative signal assessment, including the precise signal selection, the processing of multiple signals and the determination of their spatial relationship to one another, has received very little attention.

Recent developments in virtual reality (VR) have led to headsets that are lightweight to wear while offering high resolution, low latency head tracking, and a large field of view. These advances make VR an attractive technology to use in biological visualization. It allows immersive three-dimensional visualization, as opposed to a three-dimensional rendering on a two-dimensional display [1]. Because the visualizations are based on a true three-dimensional awareness of the sample, they offer an unambiguous representation as well as a more intuitive process of interaction. This can aid the process of scientific investigation and discovery.

One important factor that has limited the usefulness of microscopy volume visualization in the past was the low rendering speed. Just seven years ago it was challenging to exceed 20 frames per second (10 frames per second per eye) on consumer computing equipment [1, 2]. This is far from adequate for VR, which requires a frame rate of at least 60 frames per second per eye in order to achieve an immersive experience [3]. If this frame rate is not maintained, simulation sickness may result [4]. With recent improvements in consumer graphics performance, the frame rates necessary for VR can be achieved with reasonable rendering quality.

We present a system for visualizing three-dimensional biological microscopy data using VR. We further apply our system to the task of colocalization analysis, which is an important tool in biological microscopy.

When using VR for visualization, traditional interaction methods such as a keyboard and mouse may no longer be appropriate. We therefore consider two alternatives: the use of a gamepad, in combination with the VR headset’s head tracking, and the use of hand tracking for gesture control.

To assess the quality, suitability and characteristics of our system, a fluorescence-based three-dimensional biological sample acquired from a confocal microscope was used.

### Confocal microscopy

The confocal microscope is one of the major imaging tools used in molecular life sciences. It utilizes lasers and complex illumination settings to excite a fluorescent reagent or dye (also called a probe) within a particular focal plane and at a certain depth in the z-dimension. This avoids the collection of out of focus light that would originate from above and below the focal plane. Unlike an epifluorescence system, where the whole sample is illuminated at once, confocal microscopy makes use of a pinhole, allowing tight control of illumination in the z-dimension. By exciting the fluorescent reagents at different depths a z-stack micrographs, or images, are generated. These two-dimensional slice images can then be reconstructed three-dimensionally using direct volume rendering.

### Direct volume rendering

Direct volume rendering (DVR) is a technique that generates visualized images from a 3D volumetric data set, in our case z-stacks obtained by confocal microscopy. The z-stack data is loaded into a 3D texture map on the GPU where it is rendered, without explicitly extracting surface geometry from the data [5]. This stands in contrast to indirect volume, rendering where the surface geometry mesh is extracted before rendering takes place. Recent advances in graphics hardware make real-time direct volume rendering possible. This is not only necessary for acceptable virtual reality rendering, but also greatly improves the interaction with the sample, since it allows parameters to be changed interactively.

We implemented two DVR techniques, namely texture-based volume rendering and volume ray casting using Unity [6] with custom shaders. Each of these methods allow for different functionality in terms of the visualization.

#### Texture-based volume rendering

Texture-based volume rendering is an *object-order* approach, which means that the rendering algorithm iterates over arbitrary object slices, and in turn over the fragments on each slice, to create the visualization. These slices can also be defined arbitrarily, in the form of proxy geometry, which can be rendered instead of the image slices constituting the volume data. We aligned these slices with the viewer, where the proxy geometry is defined to be perpendicular to the viewing direction [7, 8]. These proxy geometry slices are then rendered by the GPU in a back-to-front order, during which trilinear interpolation of the volume data is applied over the interior of the proxy geometry [7]. Finally, these semi-transparent slices are blended together in order to visualize the volume data.

#### Volume ray casting

Volume ray casting is an *image-order* volume rendering technique. This means that the rendering algorithm iterates over the pixels of the final rendered image to produce the visualization of the data [9]. Practically this is achieved by representing the volume with a bounding box, and projecting rays into this volume for every visible fragment on the faces of the bounding box. The volume data is then resampled at regular discrete positions along the ray, and blended to determine the final displayed pixel. All ray casting calculations can be performed in a fragment shader, running on the GPU [7, 8]. We implemented both the standard ray casting, which produces a similar visualization as the texture-based approach, as well as pseudo-isosurface ray casting, which produces results similar to those achieved by the marching cubes and marching tetrahedra algorithms [10, 11].

#### Comparison of volume rendering techniques

Even though both texture-based volume rendering and volume ray casting can produce similar results, there are differences which justifies two implementations.

Texture-based volume rendering allows the user to move into the volume and still see the visualization, which is not possible with volume ray casting since the rendering is done on the faces of the bounding box. Hence the visualization disappears as soon as the user moves inside the bounding box. By allowing visualization from within the rendered volume, texture-based approach allows a more immersive sample investigation.

Volume ray casting, on the other hand, generally produces a sharper rendered image. Furthermore, it allows pseudo-isosurface rendering, which makes a more detailed investigation of sample regions with similar intensity values possible. This is, for example, common at cell boundaries.

### Colocalization metrics

In fluorescence microscopy, colocalization refers to two probes, or color channels, that codistribute with one another. This can be used to determine whether two molecules associate with the same structure. Colocalization can occur in two ways: co-occurance and correlation. *Co-occurrence*, is the simple spatial overlap of two probes, which are not proportional. *Correlation*, refers to two probes that not only overlap but also codistribute in proportion to one another within and between structures [12].

There are several metrics that can determine whether two probes, or color channels, merely co-occur or whether they are also correlated. We implemented a selection of such metrics, as commonly used in colocalization analysis. In order to determine co-occurrence, both *Manders’ colocalization coefficient* (MCC) and the *percentage colocalization* were calculated. The percentage colocalization is calculated between two adjustable intensity thresholds. The lower threshold was used for all metrics and functions as a noise filter to remove irrelevant or “background” pixels, which is important for accurate colocalization analysis. For both co-occurence and correlation, *Pearson’s correlation coefficient (PCC)* and *Manders’ overlap coefficient (MOC)* were calculated [12].

### Region of interest

The region of interest (ROI) is a selected sub-volume of the complete microscopy volume sample. It is a specific part of the sample that the biologist would like to investigate while ignoring the rest. For example, the amount of colocalization in the nucleus of a cell may be of particular interest. For colocalization metrics to yield meaningful information, it is generally very important to carefully outline the region of interest.

### Methods to visualize colocalization

Instead of simply calculating colocalization, it is often more intuitively useful to visualize it. Two techniques are commonly used to achieve this. Firstly, a spatial sense of the colocalization can be obtained by replacing, or *superimposing*, the colocalized voxels with white voxels. This method is, for example, useful for identifying regions of a cell or compartments where certain molecules colocalize [12]. Secondly, the outputs of the two color channels can be represented as a *scatter plot*, where the intensity of one channel is plotted against the intensity of the other, either using the original display colors or by representing the frequency of overlap between the color intensities by pseudo-colors in the scatter plot, which aids interpretation [13]. Scatter plots allow one to determine visually how well the two color channels are correlated. They also allow the detection of compartments [12].

### Virtual reality

Virtual reality (VR) refers to a 3D computer generated environment into which the user is immersed. The user can explore and interact with the virtual objects in this environment. Modern VR is mostly implemented using a stereoscopic head-mounted display (HMD), such as the Oculus Rift [14]. The HMD presents a separate image to each eye in order to achieve 3D perception of the scene. In the case of the Oculus Rift, head position tracking is accomplished via an infrared camera, and head orientation tracking is achieved using a 3-axis gyroscope, an accelerometer and a magnetometer. This allows the environment to be updated in response to head movements and allows for an immersive experience. This immersion is further enhanced when the HMD is used in conjunction with a hand tracking device such as the Leap Motion [15], or a physical haptic input device, such as a gamepad.

### Previous work

Very little work has been published in the field of immersive microscopy visualization. In the following we briefly describe four relevant systems. As far as we are aware, no system has allowed colocalization analysis in a VR environment.

#### CAVE2 hybrid reality environment

CAVE was first developed in 1992 and upgraded to CAVE2 in 2012 at the Electronic Visualization Laboratory, University of Illinois at Chicago. It is capable of 3D visualization in general, but has been extensively applied to scientific visualization.

CAVE2 is a cylindrical system, 7.3 meters in diameter and 2.4 meters high. It consists of 72 near-seamless, off-axis-optimized passive stereo LCD panels, creating an approximately 320 degree panoramic environment for displaying information at 37 megapixels (in stereoscopic 3D) or 74 megapixels in 2D and at a horizontal visual acuity of 20/20. Users can interact with visualized objects using a tracked wand, called a navigational controller. For 3D visualizations the user wears stereoscopic glasses. The position and orientation of the user’s head and the navigational controllers are tracked with an array of 10 infrared cameras [16].

#### SkinExplorer

The SkinExplorer is a VR platform for visualizing 3D reconstructed confocal microscopy images, specifically for the investigation of skin structures [17]. The system was designed to use either a large-screen 3D display, where interactions are performed using an ART Flystick3 in combination with an ART SMARTTRACK tracking system, or a desktop 3D display, where interactions is achieved using a Microsoft Kinect in combination with a joystick or a mouse for navigation. In both cases the user wears stereoscopic glasses. Head movement is tracked using sensors attached to the glasses, and in the desktop approach the Kinect automatically tracks the face to determine the position and orientation of the eyes. A GUI displays 3D widgets alongside the visualization as well as running on a remote touch interactive device [17].

#### BRAINtrinsic

BRAINtrinsic is a web-based 3D visual analytics tool that allows users to intuitively and iteratively interact with connectome data of the human brain. The system implements a visualization platform that reconstructs connectome’s intrinsic geometry, which is the topological space as informed by brain connectivity. BRAINtrinsic was developed with VR in mind and is fully compatible with the Oculus Rift [18].

#### 3D+Time brain view

The 3D+Time Brain View system visualizes functional Magnetic Resonance Imaging (fMRI) data gathered from users exposed to unfamiliar spoken languages. The system illustrates the temporal evolution of participants’ brain activity as they are introduced to a foreign language by displaying these clusters as they change over time. This is achieved by reconstructing the fMRI data using volume ray casting and displaying it on a projected stereoscopic display, with the user wearing polarized glasses. According to the developers of this system, they have been experimenting with off-the-shelf interaction technologies such as the Microsoft Kinect and Leap Motion, although no details about their implementation could be found [19].

#### Relation to proposed system

In contrast to the CAVE2 system, our algorithms are implemented on hardware that is fairly easy to obtain and much less costly. It is therefore accessible to researchers. It is also much more portable and does not require the constriction of specialized hardware. The SkinExplorer is interesting because it has also been applied to confocal microscopy. Like the CAVE systems, it is based around stationary 3D displays. The VR headset approach we propose offers better 3D immersion. Furthermore, we allow the user’s hands to be used to manipulate the sample without the need for hand held devices.

Like the system we propose, the BRAINtrinsic system makes use of modern VR to aid analysis. However, instead of volumetric reconstruction of fMRI images, the system reconstructs connectome data. In contrast, the 3D+Time Brain View system uses volume rendering to reconstruct the fMRI images of the brain. Also in common with our system, it incorporates hand tracking. However, no information on the implementation or performance thereof could be found.

## Methods

We set out to create a system that would allow improved confocal microscopy data visualization and colocalization analysis. To achieve this goal we worked closely with domain experts to identify limitations in their current analysis capabilities as well as to verify that our prototypes were indeed functional. A main objective was to make the colocalization analysis process more intuitive. In this respect, virtual reality was a very attractive option, since it offers immersion into a three-dimensional environment that is already familiar to any user. This is an important advantage over currently prevalent methods, where the sample visualization is displayed on a two-dimensional screen, and where relative spacial positions are difficult to judge due to the lack of depth.

**Fig. 1:**
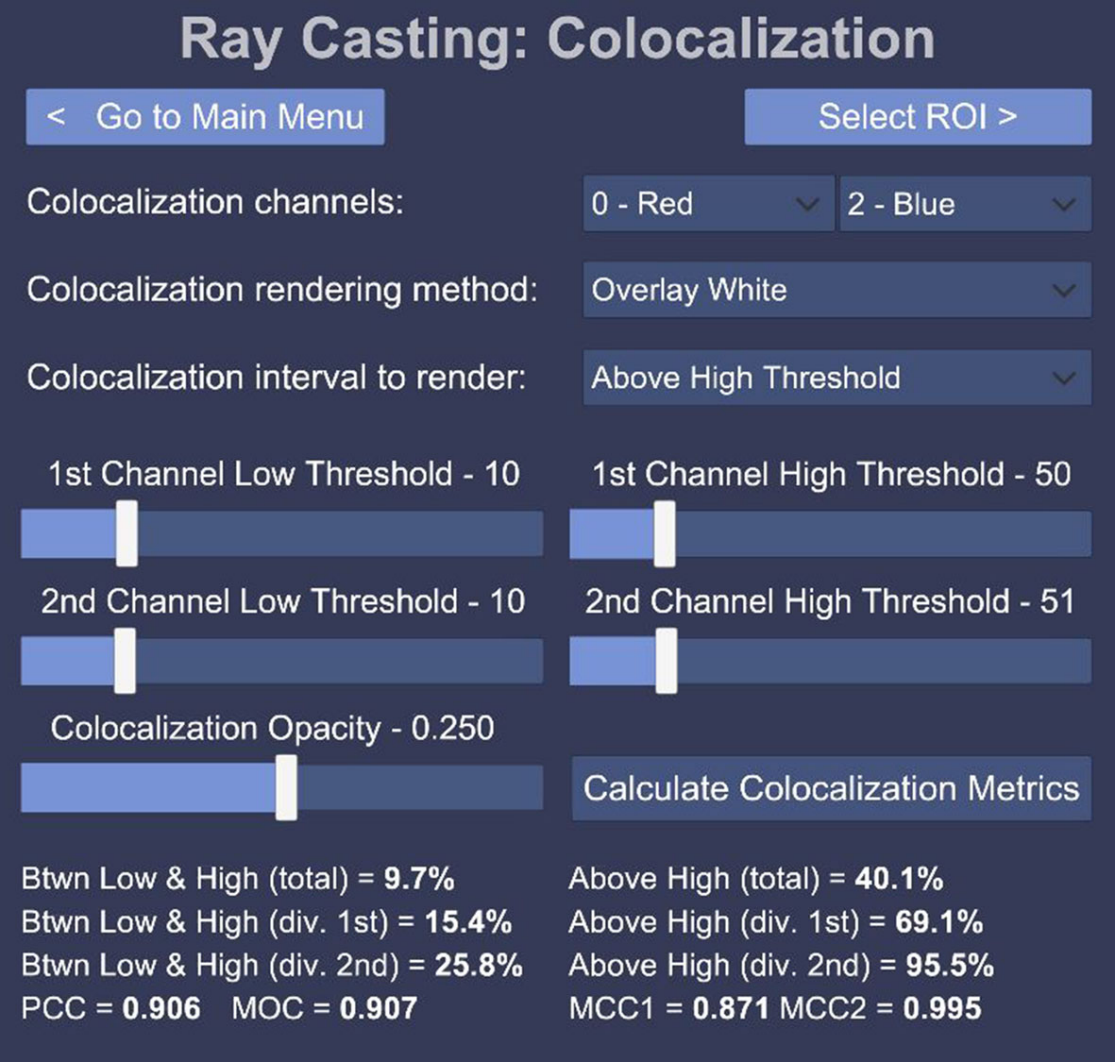
Colocalization GUI. An example graphical user interface (GUI) presented by our system. In this case the colocalization parameters can be adjusted, such as the channels that should be checked for overlap, how the rendering should be performed, what the *high* and *low* threshold should be as well as the opacity of the colocalized voxels

**Fig. 2:**
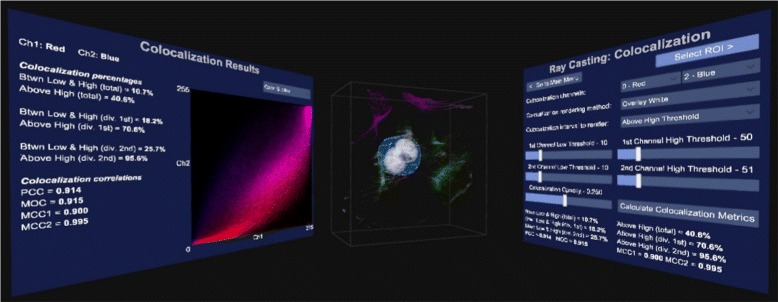
The broader GUI view. The user has GUI panels on both sides that directly face the user when looking in that direction. The colocalization GUI is to the right, the volume sample in the center and the results of the colocalization analysis to the left. The volume sample and results are updated immediately as the parameters in the GUI are varied

The user can choose to visualize the microscopy sample using either the texture-based or the volume ray casting rendering methods, depending on what they want to accomplish. In order to allow colocalization to be calculated in an interactive way, a graphical user interface (GUI) was implemented which allows the user to manipulate the rendering parameters of the sample. These parameters include the noise filtering threshold, the global opacity and the opacity of the different color channels. Further GUI panels allow more detailed colocalization analysis as well as region of interest selections. Examples of these GUI panels are shown in Figs. 1 and 2.

Our system was implemented using the Unity engine [6]. Unity uses a left-hand coordinate system, with the x-axis pointing towards the right of the view, the y-axis pointing up and the z-axis pointing away from the user. This is the coordinate system that is used for scaling the ROI.

### Sample preparation

In order to test our visualization system, mouse embryonic fibroblast (MEF) cells were stained with anti-DNA, anti tubulin and anti-actin-Alexa-633 antibody, followed by incubation with conjugated Alexa-488 donkey-anti-rabbit and Alexa-568 donkey-anti-mouse secondary antibody (Life Technologies). Nuclei were counterstained using the DNA intercalating fluorochrome Hoechst 33342. Image acquisition was performed using the Zeiss LSM 780 confocal microscope equipped with a GaAsp detector and images were acquired through z-stack acquisition, with an increment of ±0.4 *μ*m between image frames. The AxioCam MRm camera was utilized to capture images.

**Fig. 3:**
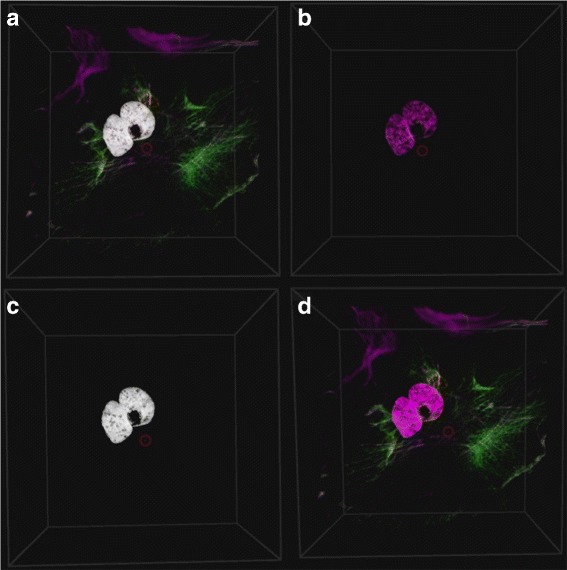
Colocalization rendering methods. The colocalization can be rendered in four different ways: **a** Overlay the colocalized voxels as *white* voxels on the original volume sample. **b** Only show the colocalized voxels. **c** Only show the colocalized voxels as *white*. **d** Don’t show any colocalization

The sample can be seen in Fig. 3 which shows a mammalian cell with 2 nuclei (blue) surrounded by small mitochondrial DNA fragments (red) as well as a thin microtubule network (green) and a thick structural actin network (magenta). Note that due to the blue and red channels overlapping, it also appears as magenta in the middle.The tubulin and actin network facilitate transport function as well as cell stability, creating a cellular skeleton.

### Input interface

When a user wears a VR headset they are visually cut off from the world. This makes traditional input such as a keyboard, mouse, pen or touch interface impractical. Two alternative methods for interacting with the system were therefore implemented. The first uses the Leap Motion hand tracking system [15], which allows the user’s hands to be visible inside the VR environment. The user can therefore use his or her hands to interact with the GUI and the sample itself in an already familiar and intuitive way. While this input method has the important advantage of absolute intuitiveness, occasionally users struggled to use the interface due to inaccuracies in the hand tracking process. This was compounded by the lack of haptic feedback that would be present when touching real objects.

As a second input method we combined the VR headset’s built-in head tracking with a traditional gamepad. Since a gamepad is a physical device that the user can hold, it allows for finer control over the visualization. Button presses are combined with the direction of the user’s gaze, as determined from the HMD’s head tracking system. Most gamepads also provide force feedback, which gives the user a greater sense of physical interaction. However, a gamepad is less intuitive and requires the user to learn which input interactions are mapped to which functions. Also, since it is not part of the rendered scene, it is less immersive.

#### Hand tracking

Hand tracking was achieved using the Leap Motion [15], which uses a stereoscopic infrared camera, along with a software API, to interpret the camera data. When attached to the front of the VR headset, the user’s hands can be tracked and rendered inside the virtual environment. Our system allows the user to translate, scale and rotate the sample using intuitive hand gestures, such as pulling and turning.

Since hand tracking is sometimes inaccurate, these gestures are designed to be simple and unambiguous. In designing suitable gestures, we have been guided by other standard VR gesture based applications designed around the Leap Motion as well as Leap Motion’s “VR Best Practices Guidelines” [20–22]. For example, we do not rely on small finger movements.

Since there is no physical feedback from the virtual content, visual feedback is used to inform the user when gestures or interactions with virtual elements are detected. We have accomplished this by rendering a bounding box around the volume sample and changing its color, based on the currently detected interaction. This was paired with optional text hints that were momentarily displayed over the sample.

In order to translate, scale or rotate the sample, the user must move both hands to be partially or totally inside the bounding box. Both hands must then perform the pinch gesture (index finger touches thumb). When this double pinch is detected, translation can be performed by moving both hands in the same direction simultaneously, while scaling can be performed by pulling the hands apart or pushing them closer together. The sample is rotated when the hands rotate relative to each other. Both rotation and translation can also be performed by pinching with only one hand, and moving the hand around for translation and turning the hand for rotation. We found that this allows for more precise rotations.

The user can interact with the GUI by touching the GUI elements in 3D space, in a similar way that a touch screen would be used.

#### Inaccuracies of the hand tracking system

The hand tracking system provided by the Leap Motion and the Orion SDK is currently the best supported consumer tracking system that also integrates easily with a virtual reality headset. It also provides the most stable hand and finger tracking that we are aware of. There are, however, several technical limitations to the system that can cause some difficulty for novice users.

Firstly, due to the use of pinch gestures for interacting with the sample, precise finger tracking is necessary. However, when the user’s hands move too far away from the tracking device, finger tracking sometimes becomes unreliable. This also makes interaction with GUI elements using a single finger difficult. These problems can, however, be mitigated by requiring the user to move physically closer to the element in question.

Secondly, because the hand tracking system needs line of sight to accurately track each finger it becomes inaccurate when the fingers are obstructed, by for example the other hand [22].

Unfortunately, neither of these problem scenarios are instinctively clear to novice users. Even when they are cautioned not to perform such problematic hand movements, the immersive nature of virtual reality makes the adherence to this advice difficult. The frequency with which these difficulties were experienced varied greatly from user to user, and it is expected that they would be largely overcome as users become experienced in using the interface.

#### Gamepad

We made use of a traditional gamepad with two analog sticks and four front facing buttons, a directional pad and four trigger buttons. Translation, scaling and rotation were performed using the analog sticks in conjunction with button presses. We combined the gamepad input with the head tracking provided by the VR headset. Using the head tracking, a 3D cursor is rendered in the center of the display. When the 3D cursor hovers over a GUI element, it is highlighted and raised slightly to indicate to the user which element they will interact with when a button is pressed on the gamepad.

Interaction with the GUI elements is accomplished by moving the 3D cursor over the element in question and pressing a button on the gamepad. When sliders are selected in the GUI their value can be changed using the direction pad. This offers an advantage over the hand tracking, since the user can change the rendering parameters without having to continue looking the GUI.

### Colocalization visualization and analysis

The GUI layout for colocalization analysis, which shows several colocalization metrics as well as scatter plots, can be seen in Figs. 1 and 2. Figure 2 shows that the GUI panels are angled to face the user when looking in that direction, with the volume sample in the center. This setup increases the immersion in the virtual environment. Colocalization is visualized in real-time as the settings are changed.

Figure 1 shows how the GUI allows the user to select the two channels that should be considered for colocalization analysis. In order to assist with the analysis the user can select to overlay the colocalized voxels on the volume sample as white voxels, or to render only the colocalized voxels either in their original colors or in white. These rendering options are illustrated in Fig. 3. Furthermore, the thresholds used in the colocalization metric calculations as well as the rendering opacity of the colocalized voxels can be adjusted. Once these parameters have been optimized interactively, the colocalization metrics and scatter plots can be calculated. All these are only calculated within a pre-selected *region of interest* (ROI), which is discussed in the next section.

### Selection of the region of interest (ROI)

**Fig. 4:**
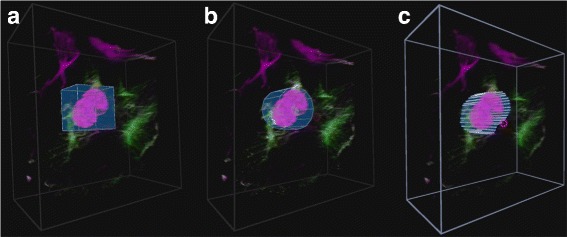
ROI selection tools. The three implemented ROI selection tools. **a** The box selection tool, **b** the *cylinder* selection tool and **c** the *freehand* selection tool. Each tool has been used to select a similar part of the volume sample

In order to effectively investigate the colocalization between two color channels, a good region of interest (ROI) selection tool is required. Three different ROI selecting tools were implemented, namely the *box*, *cylinder* and *freehand* tools. Example selections with these tools can be seen in Fig. 4. When the user is in the ROI selection mode, the same interactions used to manipulate the sample are used to manipulate the ROI. The user additionally has the ability to scale the selection along a specific axis.

When the box and cylinder tools are selected, they initially include the entire volume sample. They must subsequently be transformed to include only the section desired. When the freehand tool is selected, the user traces out the ROI using either head movement (with gamepad) or by pointing the index finger (with hand tracking). Since inaccurate freehand selections may result from the parallax effect, the volume sample is flattened and rotated to face the user before selection. Once the initial rough ROI has been selected, the user has the option to scale the ROI selection independently along any of the three axes. In the case of the freehand tool, the volume is first unflattened. By scaling along the z-axis, the user can accurately position the ROI within the volume.

After the region of interest has been selected, a ROI mask is generated in the form of a two-dimensional boolean array, as well as back and front z-positions that indicates at which depths the ROI selection starts and ends.

### User trials

Since the average user is generally unfamiliar with VR, we wanted to establish the degree of ease with which users could use our interface. More particularly we wanted to establish how difficult it is to perform certain defined manipulations using the different VR interfaces. Accordingly, user trials were carried out with a diverse group of 29 computer users. Of these, 5 were biologists that regularly work with biological visualization tools, 15 were engineers (mostly electrical and electronic engineers), another 6 were people with tertiary education in other fields and 3 had no tertiary education. Subjects were between the ages of 21 and 60. Of the participants, 20 were male and 9 female. Many statistical tests are based on the assumption that the data is normally distributed. For this reason we computed the statistical power of the task results using Matlab, to determine whether the rejection of *H*_0_ is valid. Furthermore, the power analysis proved that our sample size was adequate to ensure a statistical power of greater than 0.8 for all the tasks, except for the task to transform the sample (which was almost identical for the two interfaces) and the task to scale the ROI along the z-axis (which requires a sample size of 37).

Each participant in the study was asked to perform the same tasks, in the same order, using both the gamepad and the hand tracking interfaces. The time taken to perform each task was measured to allow subsequent objective productivity comparison. Lastly, the participants were asked to perform similar interactions with a traditional keyboard and mouse, without their time being taken, in order for the participants to gain understanding in the current standard interfaces. After using each interface, the participants were asked to complete a subjective questionnaire describing their experience when performing the different tasks.

In order to ensure that the results from different participants were comparable, all the tasks were supervised by the same researcher and all participants followed the same procedure for corresponding samples. The order in which the interfaces were tested was the same for all participants using the gamepad first, then the hand tracking and finally the traditional input. The supervising researcher ensured that all sample transformations, GUI interactions and ROI selections were completed with the same accuracy. The selection accuracies that was required for acceptance is illustrated in Fig. 4. Furthermore, the users were required to make the selection with the same accuracy between interfaces.

#### Pre-test preparation

Tests were carried out in a quiet studio environment with only the participant and the researcher present. Each user was informed about the purpose of the test and what they will see and experience. Each participant was then asked to give a subjective rating between 1 and 5 indicating their general computer proficiency, and their experience with biological visualization and colocalization analysis. Each participant also provided self-assessments of their experience in using a gamepad, a hand tracking device and a VR headset.

**Fig. 5:**
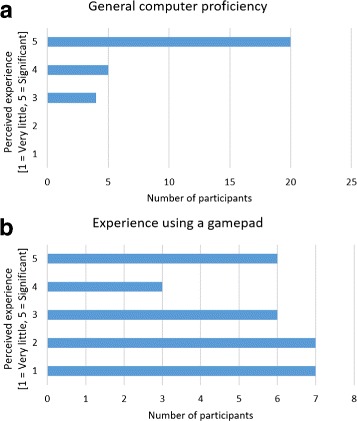
Self-assessment of computer proficiency and prior gamepad experience by the subject. **a** Participants’ general computer experience, and **b** gamepad experience

Overall the participants indicated that they had medium to high computer proficiency (Fig. 5 a). The gamepad experience among the participants was diverse (Fig. 5 b) and was a factor that we considered in our later analysis. Most of the participants had very little or no prior exposure to VR or hand tracking.

Because most participants had no prior VR or hand tracking interface experience, we used two demonstration programs to familiarize them with movement and hand interaction in a VR environment. The standard Oculus desk scene and the Blocks demo [23] created by Leap Motion were used for this purpose. Most users are astonished when using VR for the first time, and these introductions helped to ensure that the subjective feedback was based on the effectiveness and productivity of the implementation rather than the initial enthusiasm provoked by VR.

After the participant was comfortable in the VR environment, they were given a brief demonstration of the tasks that they were expected to perform, using our software, as well as an explanation of how to use the given interface. They were then given approximately 10 minutes to familiarize themselves with the interface. Only after they felt comfortable with the interface were they asked to perform the defined tasks, which are described in the next section.

#### Objective evaluation

In order to ensure a fair comparison between the participants’ experience of and productivity with each VR interface, a series of tasks was devised to ensure that the different aspects of their interactions could be tested and timed. Tasks were chosen that would cover all the aspects of the system that a biological investigator would use to perform a basic sample visualization and colocalization analysis:

1. The participants were shown an image of a desired transformation on a volume sample. In order to match this image, the participant needed to perform translation, scaling and rotation of the sample.
2. The participants were asked to change several rendering parameters to prescribed values. This mostly involved changing slider values in the GUI.
3. The participants were asked to place a ROI selection box around a prominent colocalized feature in the sample. This was divided into two steps:
  - The ROI box needed to be scaled and translated to the correct position to surround the feature.
  - Subsequently the sample needed to be rotated by 90° and the box scaled along the z-axis to match the depth of the colocalized feature.
4. Finally the participants were asked to use the freehand ROI selection tool to accurately outline the colocalized feature and adjust the z-dimension of the ROI.

Each task was explained verbally to each participant immediately before it was performed. The actions of each participant were recorded using both a video camera as well as screen capture. This allowed the time taken for each task to be accurately and unobtrusively measured later. This approach proved to be very effective in making the users feel relaxed while performing the tasks. The entire procedure took between 30 and 45 minutes for each participant. Times were measured to the closest second.

#### Subjective evaluation

After completing each task, the participants gave a perceived ease of use rating for each interface using a 5 point scale. They were also asked to provide subjective ratings describing how often they forgot the button allocations or the required input gestures. Lastly, they were asked to rate their general sense of how productive they would be when using the interface for microscopy data visualization and colocalization analysis.

Since our system was conceptualized with VR at its core, no keyboard or mouse support was implemented. In order to gain insight into how the users perceived the VR interface when compared to conventional input methods, they were asked to perform similar interactions using traditional 3D software using a keyboard and mouse, without the VR headset. The interactions were designed to mimic those implemented in standard biological applications, such as ZEN image software by ZEISS. Subsequently they were again asked to rate the the ease of use.

Finally, the participants ranked the three interfaces according to their preference for performing colocalization analysis, and to how difficult the three interfaces were to learn to use.

## Results and discussion

**Fig. 6:**
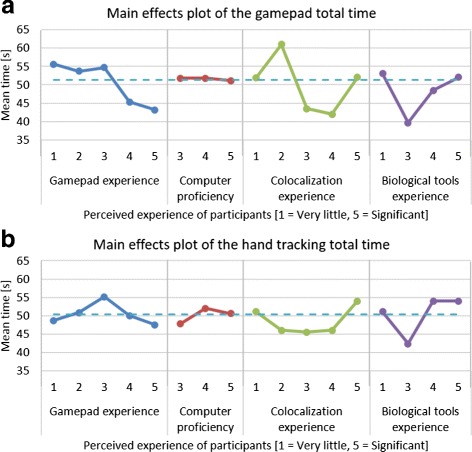
Main effects plots for time taken when using gamepad and hand tracking. The main effects plot for (**a**) the gamepad total time, and (**b**) the hand tracking total time for the four categories of self-assessed experience. The *dotted line* across the plot indicates the overall average time

Using main effects plots, which compare the means of parts of the data based on different factors, it was found that prior experience of biological visualization tools or of performing colocalization analysis had no significant influence on performance. Furthermore, the participants’ level of computer proficiency also did not affect their performance. The participants’ prior gamepad experience did however influence their performance positively in the gamepad tasks. The average total time taken to complete the required manipulations was more than 12 seconds less for users with significant gamepad experience compared to those with very little experience. The main effects plots of the total time for each interface are shown in Fig. 6.

### Subjective test results

The subjective test results were statistically analyzed using contingency tables with Pearson’s chi-square (*χ* ^2^) test to determine whether differences in participant feedback were statistically significantly (*α* = 0.05). A contingency table is a method of analyzing data in a two-way classification table and is usually used when both the dependent and the independent variables are discrete attribute data. It is a tool that can be used to test the relationship between two sources of variation [24]. Contingency tables are therefore useful when analyzing our subjective questionnaire feedback, where the independent variable is the interaction method - either the gamepad, hand tracking or keyboard and mouse, and the dependent variable is the participants’ subjective rating between 1 and 5. However, since feedback values of 1 and 2 were almost never assigned, these were grouped with the feedback value of 3, in order to allow legitimate application of Pearson’s *χ* ^2^ test [25].

By taking the average rating given for each of the tasks, it is possible to determine which interface was the preferred method of performing a certain interaction. However, these averages do not reflect whether the participants’ perception of two of the interfaces were independent. To determine whether the ratings assigned to each interface differed to a statistically significant degree, we used a *χ* ^2^ probability (*p*-value) of less than or equal to an *α* of 0.05. Furthermore, we quantified the degree of dependence using Cramér’s V, defined by:

1 $$ V = \sqrt{\frac{\chi^{2}}{N(k-1)}}   $$

In Eq. 1, *k* is the lesser of either the rows or the columns in the contingency table and *N* is the sample size [26]. The value of *V* varies between 0, indicating no association between the dependent and independent variables, and 1, for complete association between the variables. If there is high association, then the two interfaces are statistically different. Since our data has 2 degrees of freedom (*v*), we applied Yates’ correction for continuity to the *χ* ^2^ calculations before calculating *V*. In our case a *V*-value of approximately 0.4 corresponded to a *p*-value of 0.05. Therefore, V values greater than 0.4 are an indication that the two interfaces are statistically different.

**Fig. 7:**
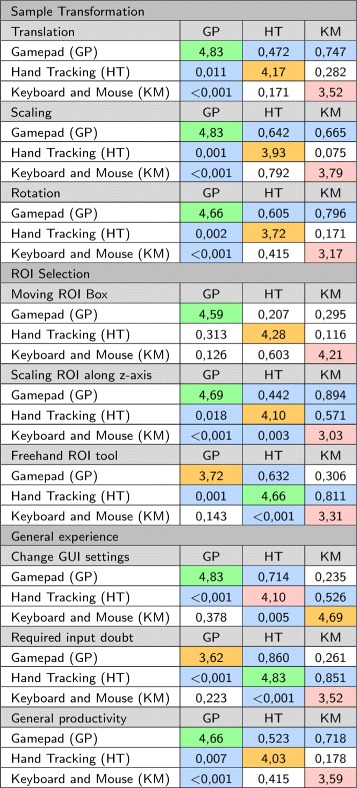
Contingency table summary of subjective results. The average rating per question is given on the diagonal. The highest average is highlighted in *green*, the second highest average is highlighted in *orange* and the lowest average is highlighted in *red*. The *p*-values are shown *below* the diagonal and the *V*-values are shown *above* the diagonal. The *p* and *V*-values that are statistically significant are highlighted

The results of the contingency table analysis are shown in Fig. 7. The average rating for the interface is shown on the diagonal, while *p*-values are shown below the diagonal and the related *V*-value are above the diagonal.

#### Discussion of subjective results

Many of the results for the hand tracking were negatively influenced by inaccuracies in the hand tracking process which are due to current technological limitations, as discussed earlier. Consequently the user experience can be expected to improve as the technology develops. This should be borne in mind when interpreting the results.

From the results in Fig. 7, for all three transformation interactions (translation, scaling and rotation) the participants gave the highest average rating to the gamepad and the lowest average rating to the traditional interface. Based on the *p*-values, the participants’ experience of the gamepad was statistically different to that of the hand tracking (all *p*-values < 0.011) and the traditional input (all *p*-values < 0.001). However, no statistically significant difference between the experience of the hand tracking and traditional input could be established (all *p*-values > 0.17).

In terms of fitting a ROI box around the colocalized area, no statistically significant preference among the different interfaces could be established (all *p*-values > 0.12). However, the ratings for scaling the box in the z-axis was significantly different for all three interfaces (all *p*-values < 0.02), with the gamepad and hand tracking being most similar (V = 0.894). In both cases the participants preferred using the gamepad, and most disliked the traditional input.

Many participants found it challenging to use the freehand ROI selection tool using the gamepad with the head tracking. On the other hand, many participants commented that this tool was significantly easier to use with the hand tracking. This is also reflected in the average rating (gamepad $\overline {x} = 3.72$; hand tracking $\overline {x} = 4.66$), where the hand tracking was the preferred interaction method, with the gamepad being the second preferred interface. From the *V*-values, it is also clear that the hand tracking was experienced significantly different to both the gamepad (V = 0.632) and the traditional input (V = 0.811). Most users were very frustrated with the inaccuracy of using a mouse to draw the ROI.

Our GUI is based on traditional sliders and buttons, which are familiar from mouse interactions. When the participants were asked to change rendering parameters using the GUI, the gamepad received a higher average rating than the traditional input (gamepad $\overline {x} = 4.83$; traditional input $\overline {x} = 4.69$). However, this difference was not statistically significantly (p = 0.378). The perception of the hand tracking was statistically significantly worse than the gamepad (*p* < 0.001) and traditional input (*p* = 0.005), with an average of 4.10. Participants felt that interacting with GUI elements using the hand tracking was cumbersome, mainly because it required very precise interactions which were challenging due to inaccuracies in the hand tracking. Furthermore, interacting with GUI elements required the user to hold their hand in the air for long periods of time. This is because the GUI remains stationary relative to the origin in the virtual environment, if the user moved a bit away from the GUI, they would either have to interact with their arms outstretched or first move closer to the GUI. Many users reported that their arms became fatigued after about 10 minutes of use. This indicates that further work in developing GUI interactions that are more suitable for VR would be beneficial. In order to allow direct comparisons, we designed our GUI to be accessible using with both gesture and gamepad interfaces. A gesture-only system need not be constrained in this way. One common alternative implementation for simple GUI interfaces is to attach the GUI to one hand and interact with it using the other hand. Our GUI was too complex for this design to be practical, however.

When the participants were asked how frequently they forgot which interaction (button press or hand gesture) performed which function, as might be expected, most felt that they never forgot the required interaction for the hand tracking. In fact it was often commented that the hand tracking was very intuitive to use and required almost no explanation. However, since button allocations had to be memorized for both the gamepad and traditional input, the initial cognitive load was greater and users had a similar experience with both. It was, however, generally felt that if either the gamepad or traditional interfaces were used for a few hours, the button allocations would be remembered instinctively. Therefore, in the long term, the intuitiveness of the hand tracking may not remain a significant advantaged.

Lastly, when the participants were asked how productive they thought they would be when performing microscopy data visualization and colocalization analysis with each interface, the gamepad received the highest average rating (4.66) with the hand tracking receiving the second highest (4.03). The main reason furnished for this response was the perceived inaccuracies in the hand tracking (described earlier), which made it difficult to use for some users.

#### Subjective interface rankings

**Fig. 8:**
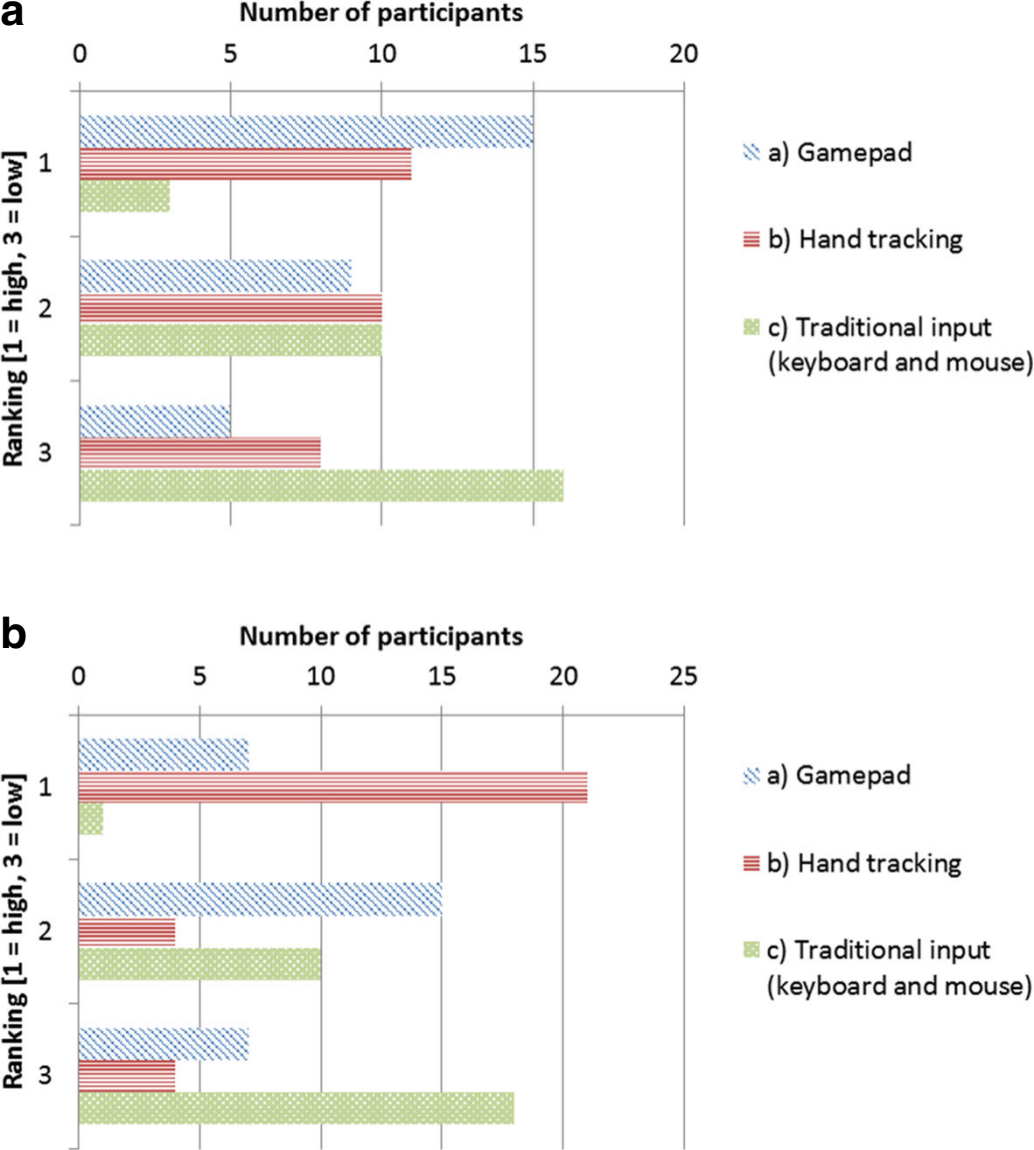
Subjective ranking of interfaces. **a** The preferred interface to use for colocalization analysis, and **b** the easiest interface to learn to use

When the participants were asked which interface they would prefer for colocalization analysis, 15 indicated the gamepad, 11 indicated hand tracking and the remaining 3 indicated traditional input, of which 2 were biologists, mainly because of its familiarity. A summary of the rankings is shown in Fig. 8 a. A recurring reason that was given to explain why the hand tracking was not the preferred choice was that the hand tracking system was too inaccurate for fine interactions, which made certain tasks more challenging than they should be. A general feeling was that if the hand tracking was improved, it might be preferred.

The participants were also asked about their perception of how easy each interface would be to learn to use. 21 of the participants felt that the hand tracking was the easiest to learn, since it was the most intuitive to use. 7 participants experienced the gamepad interface as the easiest to learn and one person preferred the keyboard and mouse, due to its familiarity. This does however show that the gamepad is perceived to be easier to learn than an already familiar interface. A summary of these rankings is shown in Fig. 8 b.

### Objective test results

**Fig. 9:**
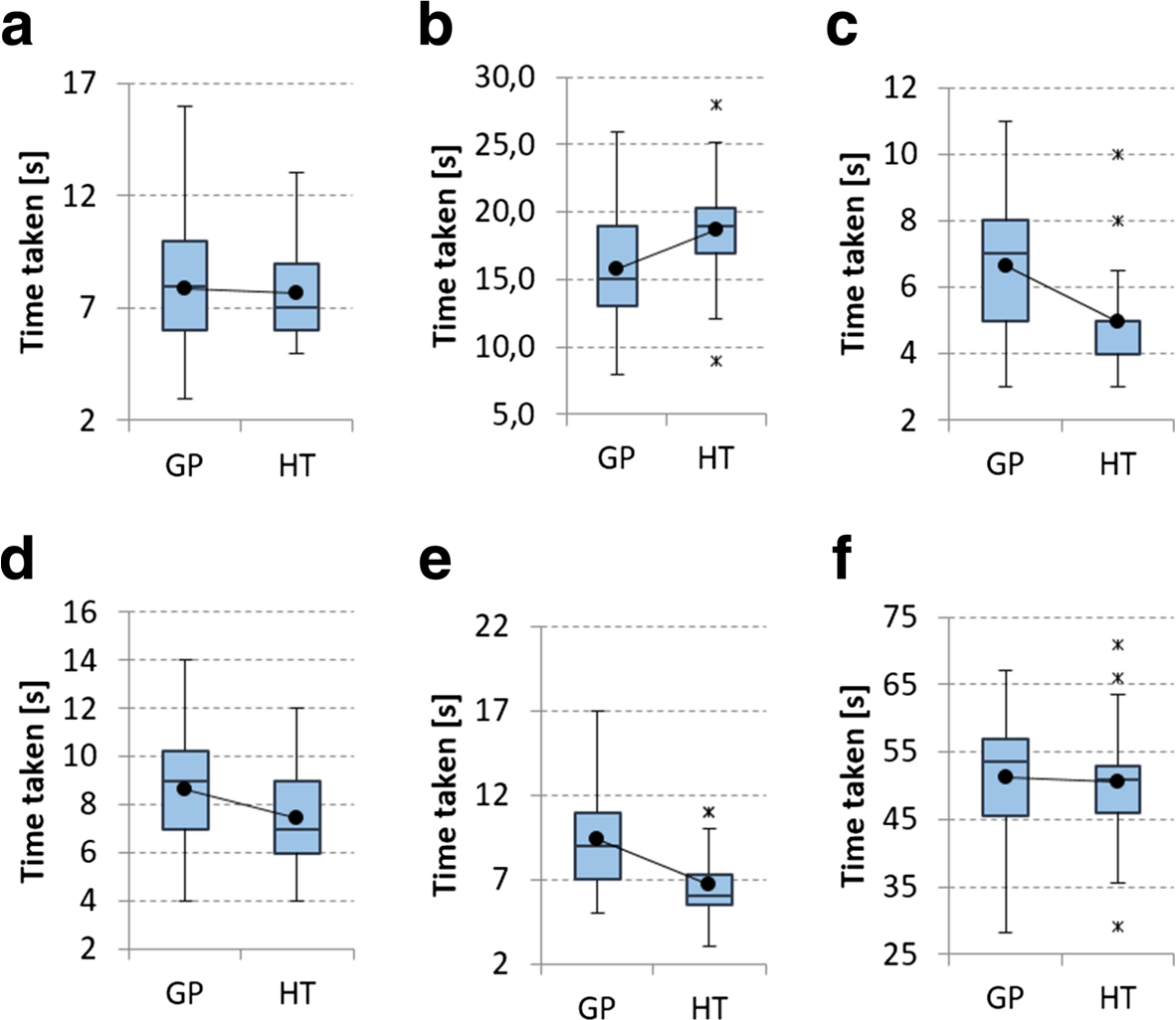
**a** Transforming the sample; **b** Changing GUI settings; **c** Moving ROI box; **d** Scaling ROI box in the z-axis; **e** Using the freehand ROI tool; **f** Total time taken. Comparative *box plots* for objective tests. *Box plots* for the objective tests that comparing the gamepad (GP) and hand tracking (HT) interfaces

The time the participants took to perform each task was statistically analyzed using the t-test. The t-test is used to determine whether two sets of data are significantly different from each other. The results of its application are shown in Table 1. Comparative box plots for the two interfaces for all the tasks are shown in Fig. 9. The t-test is based on the assumption that the data are normally distributed. Using the Anderson-Darling normality test it was determined that all gamepad tests were normally distributed (*p* >0.05). Interestingly, except for the tasks in which the parameters were changed using the GUI and in which the ROI box was scaled along the z-axis, none of the hand tracking timing results were normally distributed (*p* <0.05). However, the total time for all the tasks using the hand tracking was again normally distributed. The deviation from the normal distribution of some hand tracking tasks can largely be explained by delays caused by inaccuracies in the hand tracking itself. This means that the participants did not all share the same experience. Therefore, the t-test results that compare the gamepad and hand tracking should be regarded with some caution, even though the t-test is known to be robust also for non-normal data [27].

**Table 1:** Comparison of objective results

	Gamepad		Hand tracking			
Task	Mean	***σ***	Mean	***σ***	t-test (p-val)	Power (1- *β*)
Transforming the sample	7,9	2,8	7,7	2,0	0,38	0,0655
Changing GUI settings	**15,8**	**3,7**	18,7	3,9	**<0,01**	0,9966
Moving ROI box	6,7	1,9	**5,0**	**1,4**	**<0,01**	0,9992
Scaling ROI along z-axis	8,7	2,5	**7,4**	**2,1**	**0,03**	0,6973
Freehand ROI tool	9,4	2,7	**6,7**	**2,1**	**<0,01**	0,9800
Total time taken	48,5	9,7	45,5	6,7	0,1	0,3398

The average times and standard deviation for all the objective tasks for both interfaces are shown, as well as the statistical power values for each task. The *p*-value from the t-test indicates a statistically significant difference between interfaces when *p* <0.05. All statistically significant are shown in bold

#### Discussion of objective results

For all tasks, the average time taken to perform the required action was shorter for the hand tracking than for the gamepad, by a cumulative average of 3 seconds. Only the task in which the participants were asked to change the rendering parameters using the GUI did the gamepad perform better by an average of 2.9 seconds. As stated earlier, the inaccuracies in the hand tracking as well as GUI elements that are not optimized for hand interactions led many users to experience gesture interaction with the GUI as cumbersome.

Since the participants used the interfaces in the same order, a learning effect, in which participants became more familiar with the visualization tools and tasks, is in principle possible. This could, in turn, have contributed to the improved performance of the hand tracking. However, the hand tracking and gamepad interfaces differ vastly in how interactions are performed and each participant were thoroughly familiarized with the hardware and software environment before the test. Hence we do not believe that the average times we measured are significantly influenced by a learning effect.

Based on the *p*-value associated with the t-test, the gamepad provided a benefit when changing the GUI settings (*p* < 0.01). When moving the ROI box (*p* < 0.01) and using the freehand tool (*p* < 0.01) the hand tracking performed statistically significantly better than the gamepad. Even though the task of scaling the ROI along the z-axis indicates differences between the interfaces (*p* = 0.03), this was inconclusive since there is a Beta-risk of 30%. There is also no clear benefit to using either interface for the task of transforming the sample (*p* = 0.38). This is also suggested by the similar average times (gamepad $\overline {x} = 7.9$; hand tracking $\overline {x} = 7.7$), as well as the low statistical power of 0.0655.

From Table 1, the standard deviation of all timing results was smaller for the hand tracking than for the gamepad, except for the GUI interaction task. This can largely be ascribed to the fact that the hand tracking was more intuitive to use and was quickly learnt. On the other hand, the large standard deviation for the gamepad indicates that some participants were able to use the gamepad more productively with less training than others. This may be a result of prior gamepad experience.

#### Influence of prior gamepad experience

In terms of prior gamepad experience, the participants can be divided into two groups - those who are experienced in using a gamepad and those who are not. There were 14 participants that gave a low rating (1 or 2) for gamepad experience and 15 participants that gave a high rating (3, 4 or 5). In order to determine to what degree gamepad experience influenced performance, a brief comparison was made between these two groups.

**Fig. 10:**
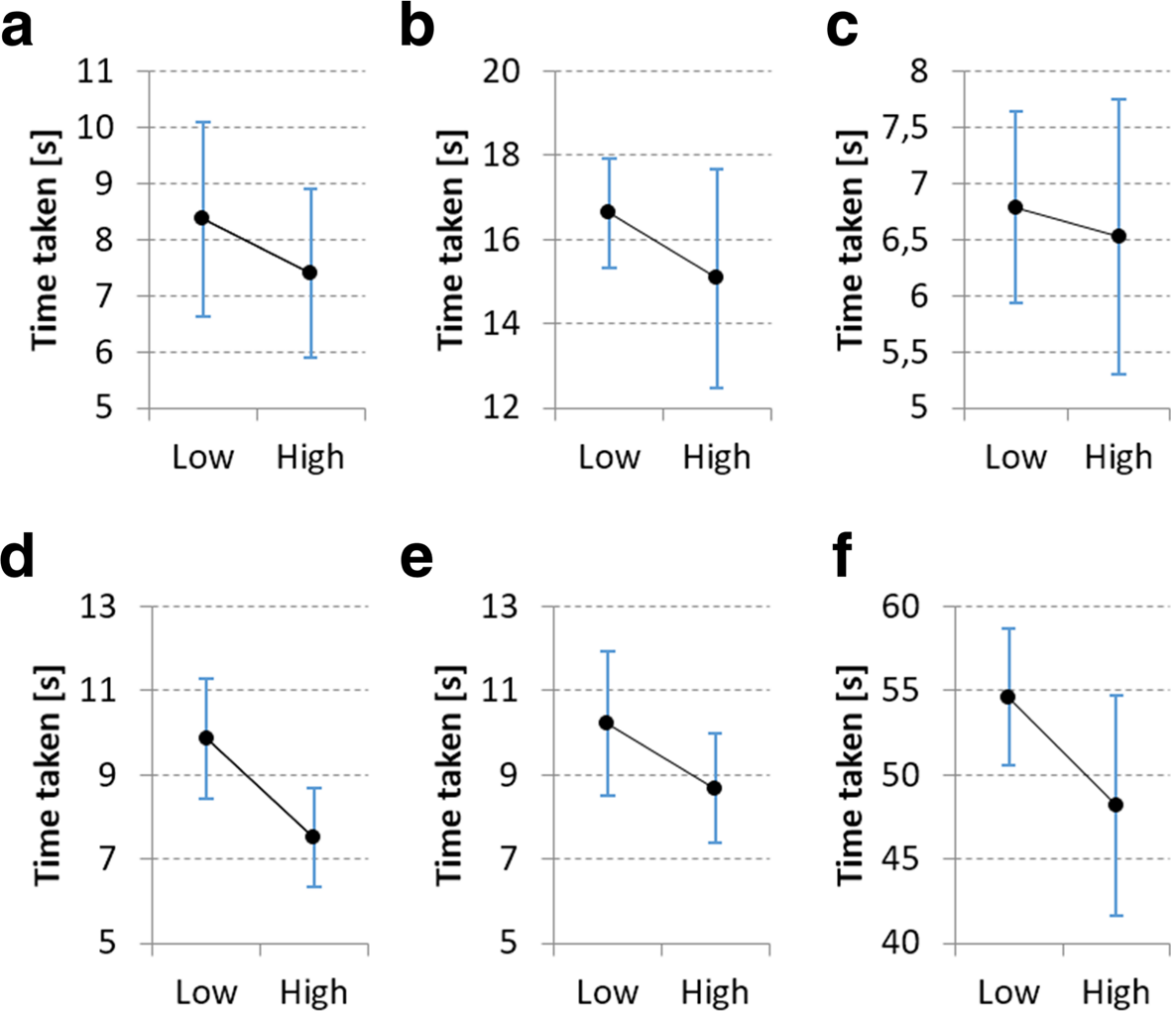
**a** Transforming the sample; **b** Changing GUI settings; **c** Moving ROI box; **d** Scaling ROI box in the z-axis; **e** Using the freehand ROI tool; **f** Total time taken. Comparative interval plots for *low* and *high* gamepad experience, showing the mean and 95% confidence intervals. Participants that gave a gamepad experience rating of 1 or 2 were considered to have *low* experience, while a rating of 3, 4 or 5 were considered as *high* experience

From the analysis it became clear that, on average, participants with high gamepad experience performed better for every task when using the gamepad than those with low gamepad experience. However, except for the task in which the ROI box was scaled along the z-axis, none of these differences are statistically significant (all *p*-values > 0.065 except for scaling the ROI along the z-axis *p* = 0.005). Comparative interval plots for gamepad usage are shown in Fig. 10. From these plots it is also clear that high gamepad experience did not consistently decrease the variability in the data. Furthermore, the overall standard deviation is significantly higher (*σ*=11.85) for users with high gamepad experience than those with low gamepad experience (*σ*=7.07). This indicates that not all users received the same productivity benefit from familiarity with this input devices. In general there seems to be no conclusive influence of gamepad experience on hand tracking performance.

### Contribution of system to improved analysis

The user perception in data handling is crucial to sample control, and can allow subsequent enhanced analysis selectivity. In colocalization analysis, it is particularly true for maximizing control over ROI selection and the associated thresholds. Often such data analysis is not performed properly due to challenges in working with the available software tools. Our system aims to allow better visualization and manipulation of colocalization data in order to improve such analysis.

The user study indicates that our system successfully offers ease of use of powerful analysis tools. This has the potential to improve the quality of colocalization analysis.

## Conclusions

We have presented a system for visualizing three-dimensional microscopy data in a virtual reality (VR) environment. As a specific objective we have focused on the calculation and analysis of colocalization within this VR environment. Our system offers fully-immersive manipulation of the microscopy data and analysis tools by means of an intuitive hand gesture interface. As an alternative method of input, a conventional gamepad was also employed. A set of user trials was performed to determine the effectiveness of our system when performing colocalization analysis and related manipulations in the VR environment (see Additional file 1: Movie S1 at http://tinyurl.com/VR-coloc).

Overall, users were very enthusiastic about the practical possibilities of the system. Interviews conducted with the five biological experts among these users, indicated a strong conviction that this system would greatly benefit them in their current work in terms of insight into the sample, as well as analysis productivity. Objective measurements show that, for most tasks, users were most productive, to a statistically significant degree, using the hand tracking interface. Despite this, the subjective assessments indicated that most users were under the impression that they were more productive using the gamepad. This negative subjective bias towards the hand tracking is ascribed to current inaccuracies in the hand tracking process, which often led to frustration. It is likely that, as hand tracking technology improves, this bias will diminish and hand tracking could become the preferred option for interaction in the VR environment. Indeed, the user trials showed that hand tracking is already perceived as the most intuitive method ofinteraction.

A secondary conclusion is that more research is required into the design of effective graphical user interface (GUI) for hand gesture input in a VR environment. The familiar offering of buttons and sliders was found by many users to be cumbersome, difficult to use with precision, and sometimes leading to fatigue.

We are also in the process of applying our system to the analysis of new biological samples, in order to determine the extent to which VR visualization offers new means to aid research.

## Additional file

Additional file 1
**Movie S1**. Virtual reality assisted microscopy data visualization and colocalization analysis. https://www.dropbox.com/s/9jb2kfuwyd2qma5/vid.mp4?dl=0. (MP4 33280 kb)

## Abbreviations

2D: Two-dimensional
3D: Three-dimensional
API: Application program interface
DVR: Direct volume rendering
GPU: Graphics processing unit
GUI: Graphical user interface
HMD: Head mounted display
IR: Infrared
MCC: Manders’ colocalization coefficient
MOC: Manders’ overlap coefficient
PCC: Pearson’s correlation coefficient
ROI: Region of interest
VR: Virtual reality
